# American Dental Association

**Published:** 1892-10

**Authors:** 


### AMERICAN DENTAL ASSOCIATION.

*First Day.—Evening Session.*

Meeting called to order by the President, Dr. W. W. Walker.

There being no miscellaneous business, Section VII., Anatomy, Pathology, and Surgery, rendered its report through its chairman, Dr. T. W. Brophy, an abstract of which report follows:

Relative to articles which first appeared in medical journals, and afterwards were reprinted in dental journals, the name of the journal will be given so that members may have easy access to them. Articles have appeared in periodicals on the following subjects on anatomy, pathology, and surgery.

"Maxillary Cysts connected with the Teeth." By Ludwig Hegstone. *Dental Review*, September number.

"Necrosis of the Maxillary Bones: A Sequence of Measles." By Dr. McCarthy, Dubuque. *Dental Review*, September, 1891.

"Affections of the Salivary Glands and Tissues in Close Proximity to them." By Dr. T. W. Brophy, December, 1891.

"A Brief Treatise on the Common Diseases of the Maxillary Sinus." By Dr. Schuhmann, March, 1892.

"Recent Contributions to the Diagnosis and Treatment of Empyema of the Maxillary." By Dr. Morgenthau. *Dental Review*, April, 1892.

"Notes on the Anatomy, Physiology, and Pathological Significance of Pain, and the Nervous Phenomena in Relation to Dental Diseases." By Dr. Gaddes. *Dental Review*, July, 1891.

"Necrosis: Its Microscopical Appearance, Causes, and Treatment." By Dr. D. M. Sabater. *Dental Cosmos*, 1891.

"The Human Mouth as a Focus of Infection." By Dr. Miller, of Berlin. *Dental Cosmos*, September, 1891.

"Diseases of the Oral Mucous Membrane." By Dr. Patterson. *Dental Cosmos*, 1891.

"Senile Atrophy of the Upper Jaw." By Drs. Abbott and Heitzmann. *Dental Cosmos*, January, 1892.

"Pathology of Trifacial Nerve." By Dr. Freeman. *Dental Cosmos*, 1892.

"Studies on the Anatomy and Pathology of the Tusks of Elephants." By Dr. Miller. *Dental Cosmos*, 1891.

"Dental Devices for correcting Deformity attending the Extirpation of the Left Half of the Inferior Maxilla." By Dr. Westlake. *Dental Cosmos*, 1891.

"Fracture of the Maxilla by a Gun-Shot Wound." By Dr. Patterson. *Dental Cosmos*, 1891.

"Where Dentistry looks over into Oral Surgery." By Dr. Curtis. *Dental Cosmos*, March, 1891.

"Two Cases of the Removal of the Gasserian Ganglion." By Dr. Andrews, of Chicago. *Dental Review*, May, 1892.

"Abscess of the Antrum, with Causes and Treatment. By Dr. Wilson, of Burlington. *Dental Review*, 1892.

"Lingual Ulceration of an Epitheliomatous Appearance due to Upper Full Artificial Denture." By Dr. A. C. Hugenschmidt, of Paris. *Dental Review*, March, 1892.

And many others, which will be printed in full with the publication of the report of this section.

The following papers and reports will be presented by the section:

"Pyorrhœa Alveolaris and its Treatment," by Dr. Cravens, of Indiana.

"The Grinding Teeth of the Herbivorous Mammalia," by Dr. A. H. Thompson, of Topeka, Kansas.

"A Report of a New Operation for the Excision of the Inferior Dental Nerve," by Dr. Cryer.

"A Study of the Molar Teeth of the Proboscidium, with Specimens," by Dr. Barrett, of Buffalo.

Report accepted.

Dr. Cravens then read the following paper:

### PYORRHŒA ALVEOLARIS: A CASE IN PRACTICE.

BY JUNIUS E. CRAVENS, D.D.S., INDIANAPOLIS, IND.

The patient, male, aged fifty years, robust, and apparently in good health, but at that time under treatment for affection of nose and throat.

*Diagnosis.*—Gums generally red and angry-looking, bleeding easily and freely; expressed considerable pus from about the anterior eight teeth in each jaw, the festoons between which were swollen, and of a violet color; complained of heat and prickling pains about the gums around the teeth specified; discovered pus on the approximal faces of roots of the pus-discharging cases; some of the roots were coated with tartar.

*July* 18.—Removed incrustations from roots in pockets, and trimmed margins of affected cases as far as practicable; washed out pockets with hot water, and treated the pockets carefully with dilute sulphuric acid (one part acid to ten parts water); directed the use of pulverized sulphur as a dentifrice morning and night, and to avoid the use of soap or other alkalies in the mouth.

*July* 19.—Gums less congested, otherwise no improvement; washed pockets with hot water, and treated them with dilute sulphuric acid.

*July* 20.—The superior first bicuspid of the right side was free of pus; gums looked and felt better; washed with hot water as usual, and renewed the treatment with sulphuric acid; advised ice applications to gums.

*July* 21.—No change; still some pus from all pockets except superior first bicuspid on the right side; decided to change treatment; washed out with hot water, as above, and treated pockets with a ten-per-cent. solution of nitrate of silver. No caustic action observed, and no pain from the lunar-caustic solution.

*July* 22.—No pus expressed except from between lower centrals and right inferior cuspid, and from between and around the superior cuspid and lateral on the right side, at which points were the deeper pockets originally; washed out pockets, and treated with nitrate of silver as above; gums looking well.

*July* 25.—Congestion gone, except slight violet tint over roots of inferior centrals, right inferior cuspid, and right superior lateral incisor, and very slight evidence of pus from pockets of the three places named. No pus observed from other points; washed out all pockets and renewed treatment with a ten-per-cent. solution of nitrate of silver.

*July* 26.—No pus whatever, except minute quantity from one side of right superior lateral incisor; gums so completely free from swelling that I was able to discover and remove a few remnants of calculus; washed with hot water, and treated with solution of nitrate of silver as above.

*July* 27.—No pus found at any point; gums apparently healthy and patient reported entire comfort; washed out and treated as above.

*July* 29.—After two days' rest found no pus at all, the pockets evidently filling up by adhesion; washed out with hot water, and treated pockets with the ten-per-cent. solution of nitrate of silver as above.

NOTE.—The solution of nitrate of silver caused a slight brownish discoloratlon about the necks of the teeth treated, but exhibited no caustic energy whatever. I think the astringent and antiseptic effects of the remedy were aided and emphasized by the thorough washing out of the pockets at each sitting with hot water. I believe the preliminary use of dilute sulphuric acid aided in removing small particles of calcareous matter from the pockets and stimulated the tendency to granulation there.

This case has an unusual interest for me, in that it appears to be cured, and in an incredibly short time. It will be under my observation for some weeks longer.

### DISCUSSION.

*Dr. Barrett.*—It seems to me that the subject is of too great importance for the paper to be passed over without discussion. If the subject of pyorrhœa alveolaris, so called, should awaken no thought in the minds of those present, it will show a very small degree of reflection on our part. You will all agree with me when I say that there seems no disease which falls within our province to treat, which calls forth so great a display of knowledge as pyorrhœa alveolaris. The etiology of the disease has never been fully comprehended; it has never, at least, been fully expressed. We have had all sorts of treatment recommended for it, sometimes with good effect, especially in the papers that are read, in the discussions that ensue, and in the statements of different practitioners. Almost invariably they have met with success. We have had recorded comparatively few failures, and yet when we endeavor to follow the same line of practice we have nearly always met with failure. At least, such has been my experience, and such has been the case with most of the men I know. Whether pyorrhœa alveolaris be solely and entirely a local manifestation of a local disturbance, or a constitutional diathesis, has not yet been determined. If there be predisposing constitutional causes, mere local treatment will be useless. If it be a simple local disturbance, if there be nothing beyond the very point of infection, if it be a disease simply of the soft tissues, then local treatment will suffice for all ordinary cases. For myself, I am not satisfied with any treatment which I have tried in the past, and I have tried almost everything. I have never yet found that local treatment was sufficient in every case. In some, topical remedies have removed the difficulty, but in the many which I have had I found that mere local remedies have simply palliated the thing for the time being, but have not produced a cure. We want a radical eradication of the disease. Many instances crowd upon my mind at the present moment that I could use as illustrations. I remember cases that have been under my care for year after year. I have been able to hold the disease in subjection partially, but where it was true pyorrhœa alveolaris, in three or six months the patient would return with a new manifestation of the disease, and I found that the whole thing was breaking out again. That has been almost the invariable rule, in cases of that kind which I have attempted to treat. The remedy which is recommended here is a cautery; I know of no therapeutic effect which it has except that of cauterization. The success which Dr. Cravens has met with in this case would indicate what? From a knowledge of the remedy and its effects, it would indicate that it was a local manifestation and nothing else. In three months or more will there not be a return of this disease? If it it be a constitutional diathesis, if it be a local manifestation of a constitutional cause, it will return. Will this be a final cure? Has he simply palliated the evil symptoms, or has he reduced the inflammation without removing the cause of the disease? That is a question which will be exceedingly interesting to have answered in the future. I think he will have a return of that patient with the same manifestations that there were in the first place; I think that with anything of that kind there is not a radical cure, but only a temporary one. I would like to hear the experience of others, whether in such cases as this, from any topical remedies, a cure has been effected, and the case has not returned with exaggerated force and buoyancy.

*Dr. Rhein.*—Some of the points that the last speaker raised I coincide with very heartily, and some others I do not. After hearing the paper read, it struck me that the case was not a case of pure pyorhœa alveolaris as distinguished from what is commonly understood as false pyorrhœa, or a result of bad hygienic care of the mouth. The one great objection to the description of the case is the lack of attention to the diagnosis at the outset. The writer stated that he believed the patient to be in fairly good health. I believe it almost essential, when a case of this kind presents itself, to settle that question first of all. Is the patient in good health or is he not? We have no right to assume that he is; that is a question that we should settle, and settle it scientifically, and the best way to do so is to give the patient a thorough physical examination, by physical means and by chemical means; the latter through a chemical analysis of his urine. Assuming that the doctor's assumption was correct, that he was in fair physical health, I unhesitatingly believe that there was no real disease, as we look on it, and, coming to the question brought out by the last speaker, as to the etiology of pyorrhœa alveolaris, in my mind at the present day that question is pretty firmly established, and I believe it is established in the minds of a great many other practitioners,—to wit, that the disease which we recognize as pyorrhœa alveolaris is unquestionably either the sequence of some constitutional disturbance, or that disturbance is present at the time. That is to say, it is the result of a faulty nutrition of the parts. If that faulty nutrition is caused by some severe constitutional disturbance that cannot be permanently cured, it is very easy for us to determine the fact that the pyorrhœa cannot be cured. If it is an incurable disease, we can give a definite prognosis. If, on the other hand, it is curable, we can give a still more favorable prognosis. This is the point I cared to bring out in the discussion of the last speaker.

The nomenclature is unquestionably a very poor one, because in a strictly literal sense this is not a case of pyorrhœa alveolaris. It is a case of discharge of pus from the alveolar sockets; but we must distinguish it from the common form of a badly-kept mouth, as distinguished from a sequence of a constitutional disturbance, and what I particularly desire to call your attention to is the fact that having determined that (if we cannot do so ourselves, we can by consultation with the physician of the patient ascertain the exact condition of the patient), we can give some definite prognosis as to the result. I know that many cases have passed through my hands that have never been cured, and I have never promised to cure them, because I had found that condition of the system present when I knew the case was hopeless. The patient may have been suffering from tuberculosis, or had some trouble of the kidneys, or there may have been a gouty condition of the system which I have invariably found connected with this disease, and where we find it associated with such a grave condition as to be incurable it is useless for us to give a favorable prognosis.

*Dr. Morgan.*—My observations have led me to the belief that it is very largely an inherited disease, a local manifestation of a debilitated condition of the system. In a large majority of the cases you will find that entire families are affected more or less with this disease, so that I believe it is a constitutional disease, and if it is to be cured at all, it must be done either by constitutional treatment or by a very radical local treatment. Every case can be cured without a single exception. Just remove the tooth and nature will take care of the balance. I do not know whether I ever cured a case of decided pyorrhœa alveolaris in my life that remained permanently relieved, unless I removed the tooth. I remember an instance of six persons in one family: the children came into my office when they were from eight to fifteen years of age, and until they had passed maturity I preserved their mouths in pretty fair condition, but I treated them every six months. I had an arrangement with the parents by which I could call them to the office and give them treatment, and until they were married their teeth were all preserved intact. The trouble, to my mind, is certainly with the tissues that belong to the teeth; therefore, if you remove the teeth you get rid of the trouble.

*Dr. Harlan.*—The subject under discussion is the treatment of a single case, and not the etiology of the disease or the multiplicity of remedies. There is no objection to the use of a diluted solution of sulphuric acid around the roots of the teeth as a preliminary in the beginning of the treatment of such a case, because the diluted sulphuric acid contains about thirteen per cent. of sulphuric acid to eighty-seven per cent. of water, and then you dilute that ten times, so that when it comes in contact with the fluids of the mouth it will do no damage whatever to the tissue of the tooth. It would aid very materially, and in my opinion would act much better than a diluted solution of aromatic sulphuric acid, which is one of the worst remedies for that purpose in the entire materia medica, because it will not dissolve the calcareous deposits that are found on the roots of teeth, and diluted sulphuric acid will.

With reference to a ten-per-cent. solution of nitrate of silver, it is a very good remedy; it has been used in the treatment of conditions of the mucous membrane, but never in so concentrated a stage. What are the effects that should naturally follow the use of a ten-per-cent. solution of nitrate of silver? First, it would be a powerful astringent; secondly, it would act as a stimulant, and the reason for the checking of the pus and the closing up of the pockets in such a short space of time would be due to the properties it possesses. To say a case was cured after eleven days' treatment is something that I could hardly think possible, because if the pockets were of the depth of one-half the length of the root there could not be a reproduction of tissue in that short time with a continual and repeated washing of the pocket. The effect of the use of hot water as a preliminary is excellent, and there is nothing to say against that, because hot water is a very useful agent in many cases and much more beneficial in the vast majority of cases that dentists are called upon to treat than the agents that they do use, because many of them are used unintelligently. Hot water as a therapeutic agent is something that dentists fail to utilize, and so Dr. Cravens has made a very good point in that. I invariably use it, and whenever I desire the effect of a medicinal agent I make use of diluted sulphuric acid, but I dilute it with cinnamon-water or cassia; I think that in that way I perhaps may get a little better effect than from ordinary hydrant-water.

As the question of the treatment of pyorrhœa alveolaris will come up under the head of therapeutics, I just wish to say that I thank Dr. Cravens for his presentation of the subject in this way, because anything that will add to our resources in the treatment of a disease of this kind is very valuable. It is undoubtedly much more local than some of the gentlemen think; for, as Dr. Morgan has stated, whenever you extract the tooth, the case gets well. No one ever saw a case where the teeth were extracted that the case did not get well. There is a strong probability that it is a purely local disease, with many constitutional manifestations, most of them concomitant and not the cause.

*Dr. Cravens.*—I have very little to say in closing the subject. I think Dr. Barrett misunderstood me in regard to the caustic effect of the nitrate of silver solution. I stated in the paper distinctly that I could observe no caustic effect at all. I cannot agree with Dr. Rhein in regard to the constitutional character of the affection, or that it is complicated always with constitutional affections. Dr. Thomas W. Evans, of Paris, told me that he had seen so many cases associated with affections of the kidneys that he believed it would very likely come to be recognized as a manifestation of kidney trouble. He also told me that in that country many dentists regarded it as a form of gout.

In regard to the analysis of the urine, I do not believe the dentist can always do that. I have not the facilities, and I doubt very much if I have the ability. I would not do it if I had both. In regard to whether the case was cured or not, I simply expressed the opinion that I thought it was. I hope I will have the pleasure of hearing more about the case later on from some of the Nashville dentists who are here to-day, as the patient was a Nashville gentleman.

I want to thank Dr. Harlan for the encouraging remarks he made.

(To be continued.)

